# Clinical assessment of levamlodipine besylate combination therapy for essential hypertension: A protocol for systematic review and meta-analysis

**DOI:** 10.1097/MD.0000000000029148

**Published:** 2022-04-01

**Authors:** Guo-Yao Dai, Ye-Hua Zhu

**Affiliations:** Department of Pharmacy, General Hospital of Eastern Theater Command, Nanjing, Jiangsu Province, China.

**Keywords:** efficacy, hypertension, levamlodipine besylate, meta-analysis

## Abstract

**Background:**

Essential hypertension has been regarded a significant risk factor for cardiovascular disease across the globe, and a significant escapable causation of early death as well as morbidity in the U.S. When angiotensin II receptor blockers and calcium channel blockers are used to treat essential hypertension, most patients will have inadequate blood pressure management. As a result, including a diuretic in the regimen is necessary. The current study's aim is to investigate the effectiveness as well as safety of levamlodipine besylate combination therapy in treating essential hypertension at varying degrees of severity.

**Methods:**

In establishing the effectiveness and safety of the mix of levamlodipine besylate and dihydropyridine for essential hypertension, the authors will conduct a systematic review and, where applicable, a meta-analysis of randomized controlled clinical trials. A total of 8 electronic databases will be used in the search, including 4 English databases (PubMed, Web of Science, EMBASE, and Cochrane Library) and 4 Chinese databases (China National Knowledge Infrastructure, Chinese BioMedical Literature database, Chinese Scientific Journal database, and WanFang database). All articles published in the databases will be considered between their inception and January 18, 2022. Only articles published in English or Mandarin Chinese will be picked. A group of writers will independently evaluate each reference to see if it is eligible and whether there are any duplicates. The same authors will do data extraction for all eligible studies and use the Cochrane risk of bias tool to evaluate the risk of bias in the trials chosen for inclusion.

**Results:**

The analysis will evaluate the efficiency and level of safeness of levamlodipine besylate combined treatment for essential hypertension.

**Conclusions:**

Our systematic review will offer evidence for judging whether levamlodipine besylate combination therapy can be considered an effective intercession for essential hypertension.

**Ethics and dissemination:**

Ethical approval will not be required as no original data will be collected as part of this review.

**Registration number:**

DOI 10.17605/OSF.IO/H8ZR2.

## 1 Introduction

According to the World Health Organization, about 1 out of every 3 persons in the world has high blood pressure.^[1]^ Hypertension is identified as a primary risk factor for stroke, cardiovascular disease, and premature death worldwide, with a predicted global incidence of 31.1% in adults in 2010, a figure that has been projected to rise as the prevalence of obesity the ageing of the population increases.^[2–4]^ There were a record 85.7 million hypertension patients in the U.S. between 2011 and 2014, accounting for a third of the total adult population and two-thirds of the population aged 60 years and over. Out of which, 84% of patients were aware that they had hypertension, three-quarters of those diagnosed with hypertension were given antihypertensive medication, and only 50% of the patient's blood pressure was adequately managed.^[5]^ A 1.57-fold and a 1.74-fold rise in the risk of all-cause and cardiovascular death, respectively, compared to those treated for controlled hypertension, was seen in those who were treated uncontrolled hypertension.^[6]^ A large number of individuals with hypertension, as a result, need treatment techniques that are extremely effective, safe, and tailored to their needs. Preliminary blood pressure-lowering strategies are mostly focused on monotherapy.^[7]^

However, it is necessary to use a combination of medications in many circumstances to get the desired blood pressure levels.^[7,8]^ According to some sources, combination therapy is more successful than monotherapy.^[9,10]^ Combination therapy is available in 2 forms: a fixed-dose combination, which involves the use of a minimum of 2 active agents combined in a single pill, and a free-equivalent combination, which involves the use of the conforming medication components in a different way than the fixed-dose combination. To be sure, the free-equivalent combination is chemically equal to the combined fixed-dose combination. Many studies, however, have shown that using a fixed-dose combination has a better effect on blood pressure management while also minimizing the utilization of medical resources by enhancing the patients’ acquiescence as well as perseverance to therapy. Previous research has not systematically evaluated the efficacy and degree of safety of levamlodipine besylate combination therapy for essential hypertension using high-quality data, and this is the first time that has happened. This protocol aims to determine the effectiveness as well as safety of levamlodipine besylate combination treatment for essential hypertension.

## 2 Methods

Enrolment was completed in February 2022 on the Open Science Framework with registration number: DOI 10.17605/OSF.IO/H8ZR2. The present study has been reported following the Preferred Reporting Items for Systematic Reviews and Meta-Analysis Protocol statement guidelines.^[11]^

## 3 Eligibility criteria for study selection

### 3.1 Types of studies

Each pertinent published randomized controlled trial on levamlodipine besylate combination therapy for essential hypertension will be incorporated in the analysis. Case reports, non-randomized controlled studies, and observational trials are prohibited. The languages in which the publications are available are restricted to English and Chinese.

### 3.2 Types of participants

Regardless of gender, ethnicity, or age, participants with essential hypertension will be evaluated for inclusion in this study on a thorough basis.

### 3.3 Types of interventions

The experimental intervention entails all types of levamlodipine besylate combined therapy. The controls can be treated with any therapies other than levamlodipine besylate therapy.

### 3.4 Types of outcome measures

The following results will be measured: medication compliance, medication adherence, systolic and diastolic arterial blood pressures before and after therapy, adverse responses, and treatment safety.

## 4 Search methods for the identification of studies

### 4.1 Electronic searches

A total of 8 online-based databases will be searched, including 4 English databases (PubMed, Web of Science, EMBASE, and Cochrane Library) and 4 Chinese databases (China National Knowledge Infrastructure, Chinese BioMedical Literature database, Chinese Scientific Journal database, and WanFang database), with all articles published in the databases between their inception and January 18, 2022, is considered. When searching for literature, the writers will use medical topic headings and phrases relating to “hypertension,” “levamlodipine besylate,” and “randomized controlled trial,” among other things.

### 4.2 Searching other resources

The authors will personally scan the reference lists of linked journals and conference proceedings to ensure that no potentially eligible trial is missed.

## 5 Data collection and analysis

### 5.1 Selection of studies

Before proceeding to the second screening, 2 writers will independently review each title and abstract as well as the rest of the manuscript, rejecting any that do not seem to meet the inclusion requirements. The secondary screening will comprise reviewing the full texts of possibly eligible literature to exclude studies that are not appropriate for inclusion. All differences of opinion must be resolved via dialogue or by consulting with another independent author.

### 5.2 Data extraction and management

Two independent authors will extract the following information after the studies have been identified: sample size; gender; age; diagnosis; intercession; and treatments administered to the control cohort; comorbidities; curative effect index; outcome indicators; and adverse outcomes. All differences of opinion must be resolved via dialogue or by consulting with another independent author.

### 5.3 Assessment of risk of bias in included studies

A pair of authors will independently evaluate the quality of the methodology in all included studies using the bias risk assessment instrument suggested by the Cochrane Handbook, which consists of 7 domains and will be used by the Cochrane Collaboration. To differentiate between low bias risk and unclear bias risk, each domain will be labeled as low, unclear, and high bias risk. All differences of opinion must be resolved via dialogue or by consulting with another independent author.

### 5.4 Measures of treatment effect

In this research, the impact sizes of dichotomous data will be reported as the relative risk, and the effect sizes of continuous data will be expressed as the weighted mean differences. All metrics’ 95% confidence intervals need to be calculated and reported.

### 5.5 Dealing with missing data

We shall first contact the senior or corresponding author to gather any missing or incomplete information. If there is no answer, we will make an educated guess as to what the missing data is.

### 5.6 Assessment of heterogeneity

The heterogeneity will be analyzed quantitatively utilizing the *I*^2^ statistic, and if the *I*^2^ value is more than 50%, we will look into the cause of the heterogeneity further.

### 5.7 Assessment of reporting bias

If a significant number of randomized controlled trials (>10) are included, funnel plots or Egger and Begg tests will be used to examine any conceivable publication bias.

### 5.8 Sensitivity analysis

To establish the robustness and reliability of the findings, sensitivity studies should be carried out in experiments with adequate data to be handled as required.

## 6 Discussion

Even though several researchers have stated that the fixed-dose combination may have a beneficial influence on blood pressure control while simultaneously ascertaining a reduction in medical resources by improving patients’ dependency and perseverance with treatment, more research is needed to confirm this. The effectiveness and degree of safety of levamlodipine besylate combination treatment for essential hypertension, on the other hand, have not been addressed in prior systematic review topic is still at the safety stage. A systematic review will be conducted to understand further the effectiveness and safety of levamlodipine besylate combination treatment for essential hypertension. The outcomes of this trial are intended to give the most up-to-date information on the effectiveness and safety of levamlodipine besylate combination treatment for essential hypertension, which is currently lacking. Furthermore, the findings of this research may provide important information to patients, doctors, and health policymakers.

## Author contributions

**Conceptualization:** Guo-Yao Dai, Ye-Hua Zhu.

**Data curation:** Guo-Yao Dai, Ye-Hua Zhu.

**Formal analysis:** Guo-Yao Dai.

**Funding acquisition:** Ye-Hua Zhu.

**Investigation:** Ye-Hua Zhu.

**Methodology:** Guo-Yao Dai, Ye-Hua Zhu.

**Project administration:** Guo-Yao Dai, Ye-Hua Zhu.

**Software:** Guo-Yao Dai, Ye-Hua Zhu.

**Supervision:** Guo-Yao Dai.

**Validation:** Guo-Yao Dai, Ye-Hua Zhu.

**Visualization:** Guo-Yao Dai, Ye-Hua Zhu.

**Writing – original draft:** Guo-Yao Dai.

**Writing – review & editing:** Ye-Hua Zhu.
